# Breed of origin analysis in genome-wide association studies: enhancing SNP-based insights into production traits in a commercial Brangus population

**DOI:** 10.1186/s12864-024-10465-1

**Published:** 2024-07-01

**Authors:** Gabriel A. Zayas, Eduardo Rodriguez, Aakilah Hernandez, Fernanda M. Rezende, Raluca G. Mateescu

**Affiliations:** 1 Department of Animal Sciences, University of Florida, Gainesville, FL USA; 2 Department of Animal Science, North Carolina State University, Raleigh, NC USA

**Keywords:** Angus, Brahman, Heterosis, Dominance, Breed of origin, GWAS

## Abstract

**Background:**

Carcass weight (HCW) and marbling (MARB) are critical for meat quality and market value in beef cattle. In composite breeds like Brangus, which meld the genetics of Angus and Brahman, SNP-based analyses have illuminated some genetic influences on these traits, but they fall short in fully capturing the nuanced effects of breed of origin alleles (BOA) on these traits. Focus on the impacts of BOA on phenotypic features within Brangus populations can result in a more profound understanding of the specific influences of Angus and Brahman genetics. Moreover, the consideration of BOA becomes particularly significant when evaluating dominance effects contributing to heterosis in crossbred populations. BOA provides a more comprehensive measure of heterosis due to its ability to differentiate the distinct genetic contributions originating from each parent breed. This detailed understanding of genetic effects is essential for making informed breeding decisions to optimize the benefits of heterosis in composite breeds like Brangus.

**Objective:**

This study aims to identify quantitative trait loci (QTL) influencing HCW and MARB by utilizing SNP and BOA information, incorporating additive, dominance, and overdominance effects within a multi-generational Brangus commercial herd.

**Methods:**

We analyzed phenotypic data from 1,066 genotyped Brangus steers. BOA inference was performed using LAMP-LD software using Angus and Brahman reference sets. SNP-based and BOA-based GWAS were then conducted considering additive, dominance, and overdominance models.

**Results:**

The study identified numerous QTLs for HCW and MARB. A notable QTL for HCW was associated to the *SGCB* gene, pivotal for muscle growth, and was identified solely in the BOA GWAS. Several BOA GWAS QTLs exhibited a dominance effect underscoring their importance in estimating heterosis.

**Conclusions:**

Our findings demonstrate that SNP-based methods may not detect all genetic variation affecting economically important traits in composite breeds. BOA inclusion in genomic evaluations is crucial for identifying genetic regions contributing to trait variation and for understanding the dominance value underpinning heterosis. By considering BOA, we gain a deeper understanding of genetic interactions and heterosis, which is integral to advancing breeding programs. The incorporation of BOA is recommended for comprehensive genomic evaluations to optimize trait improvements in crossbred cattle populations.

**Supplementary Information:**

The online version contains supplementary material available at 10.1186/s12864-024-10465-1.

## Background

Approximately 45% of beef cattle in the United States are located in subtropical regions, primarily spanning the southern and southeastern states. In these areas, cattle frequently face challenges posed by hot and humid climates [1]. These environmental conditions subject them to extreme heat stress, which can adversely impact their well-being, hinder growth, and reduce overall productivity. To mitigate such challenges, producers often resort to crossbreeding, integrating both taurine (*Bos t. taurus*) and indicine (*Bos t. indicus*) breeds. This strategy is aimed at capitalizing on the strengths of both subspecies, harnessing the resilience of the second while maintaining the superior meat quality of the first [2]. Central to the effectiveness of this approach is the principle of heterosis. Heterosis is the superior performance of the crossbred offsprings compared to their purebred parents. Key to this enhanced performance is the dominance effect, which allows for the advantageous combination of alleles from distinct breeds. As technology advances, the incorporation of genomic tools hold the promise of enhancing and refining the benefits of heterosis [2].

Genome wide-association studies (GWAS) using SNP data in multi-breed and crossbred beef cattle have previously identified heterotic QTL, genetic variants contributing to the expression of traits that benefit from increased vigor observed in hybrids [3]. However, a critical factor that is frequently overlooked pertains to the breed of origin of alleles (BOA). The term “BOA” specifically denotes the breed from which a particular genetic marker is inherited [4, 5]. Understanding the breed of origin is essential because it sheds light on the genetic lineage and ancestry of specific alleles, providing valuable insights into the inheritance patterns and contributing factors to observed traits. In crossbred and composite cattle, such as Brangus, it is important to note that markers that appear identical may actually come from different parent breeds (Angus or Brahman). The impact of a marker on traits can vary depending on its origin, as there are differences in linkage disequilibrium between the breeds. Hence, even though these alleles may seem alike, their impact on production traits can differ significantly depending on their distinct breed origins. When combined with information of dominance effects, identifying the BOA provides a more precise representation of genomic heterosis given that the observed heterosis in admixed populations is a consequence of heterozygous BOA. This comprehensive understanding will not only highlight breed-specific advantages but will also outline potential avenues for trait improvement, formulating customized breeding strategies that are optimal for crossbred and composite breed scenarios. Studies using BOA information for genomic prediction in dairy cattle have shown that incorporation of such information is beneficial in multi-breed production schemes [6].

The objective of this study was to conduct GWAS on hot carcass weight (HCW) and marbling (MARB) and identify genetic variants with additive, dominance, and overdominance effects using SNP and BOA information within a Brangus commercial herd. This has the potential to provide a more holistic perspective on the complex interplay of genetic factors shaping these traits of interest.

## Materials and methods

### Animals and phenotypic measurements

The research protocol was approved by the University of Florida Institutional Animal Care and Use Committee number 201,003,744. The study population included 1,066 Brangus steers from the Seminole Tribe of Florida, Inc. born in 2014 and 2015. Harvest information, sample collection and trait measurement are described in detail by Rodriguez et al. [7]. Briefly, cattle were fed at Quincey Cattle Company, a commercial feedlot located in Chiefland, Florida, where they received a conventional feedlot diet containing corn, protein, vitamins, and minerals until they attained a subcutaneous fat thickness over the ribeye of approximately 1.27 cm. Cattle were processed at a USDA-inspected inspected slaughtering facility (FPL Food LLC., Augusta, Georgia) facility following standard processing protocols by penetrating captive bolt followed by immediate exsanguination [8]. Hot carcass weight (HCW; kg) was recorded after harvest. Carcasses were ribbed between the 12th and 13th rib at 48 h postmortem and marbling score (MARB) was then evaluated according to USDA standards outlined by Hale et al. [9].

### Genotyping and quality control

Genomic DNA was extracted from either tissue or blood samples using the QIAamp DNA Mini DNA kit (Qiagen, Valencia, CA, United States) following the manufacturer’s protocol and stored at − 20 °C. Genotyping was conducted using the Bovine GGP F250 array (GeneSeek, Inc., Lincoln, NE) containing 221,115 SNPs enriched with functional variants including non-synonymous, frameshift, and stop mutations. Only autosomal SNP were mapped to the ARS-UCD1.2 assembly and retained for further analysis. Quality control (QC) filtering was performed with PLINK2 [10]. QC for the GWAS excluded animals with a genotype completion rate below 90% and markers with a minor allele frequency below 1% and a genotype call rate below 90%. This QC process resulted in 127,912 SNPs and 1,024 cattle suitable for further analysis. BOA was predicted using Local Ancestry in adMixed Populations using Linkage Disequilibrium (LAMP-LD) analysis [11, 12]. QC for LAMP-LD analysis used stricter criteria, with markers requiring a call rate over 99%. This stricter QC resulted in a set of 108,688 SNPs, and 1,024 SNPs with an allele frequency difference (AFD) of at least 5% between the purebred populations were retained, culminating in 93,751 SNPs for the LAMP-LD analysis.

### Breed of origin

LAMP-LD was used to infer percentages of local ancestry of each animal [11, 12]. LAMP-LD uses hidden Markov models of haplotype diversity of the ancestral/purebred populations within a window-based framework to trace the origin of alleles in the admixed population [11, 12]. Purebred Angus and Brahman cattle from the University of Florida’s Multibreed Angus x Brahman herd were used to represent the purebred populations for the LAMP-LD analysis. A total of 123 purebred Angus cattle and 406 purebred Brahman cattle were used as the reference population. Only markers with an AFD ≥ 5% between purebred population were used, to ensure sufficient differentiation between breeds. The local ancestry results from LAMP-LD were then used to infer the BOA. The BOA of the resulting 93,751 SNPs were then converted into a pseudo-genotype format using in-house scripts, where 0 represented homozygous Angus (AA), 1 represented the heterozygote state (AB/BA) and 2 represented homozygous Brahman (BB).

### Estimation of genetic parameters

Average information restricted maximum likelihood (AIREML) variance components were estimated using single-trait animal linear mixed models with alternative genomic kinship matrices (**G**). Our approach involved fitting distinct models for both SNP and BOA data, explicitly considering additive, dominance, or overdominance genetic effects. In this study we conducted a principal component analysis (PCA) on SNP genotypes using PLINK [10], and incorporated the first two principal components (PC1 and PC2) across all models as fixed effects. The top two PCs were included to account for some variation that would be otherwise explained by genotypes, such as subtle breed composition differences in Brangus cattle as well as some pedigree differences. Additionally, to maintain methodological consistency, PC1 and PC2 were also included in the additive genetic model. Concerns about the principal components potentially overshadowing the additive model’s variance led us to validate our approach by examining the estimated variance components with and without including the principal components in the model (Additional file 2 Table S1). Given the minimal differences observed in variance estimates and to facilitate model comparison, PC1 and PC2 were also retained in the additive models. All analyses were executed with the `airemlf90` package, which is part of the BLUPF90 software suite [13].

The employed mixed model is represented by:

$$y=\mathbf{X}b + \mathbf{Z}u+e$$

Where:

- *y* denotes a vector of phenotypic records.
- **X** and **Z** are incidence matrices, connecting phenotypic records to fixed effects and genetic effects, respectively.
- *b* is a vector of the fixed effects. This includes a categorical contemporary group, which is composed of feedlot pen nested within ranch location. Specifically, the contemporary group comprises 36 distinct levels, each containing a minimum of five individuals, ensuring adequate representation across the dataset. Additionally, the first two principal components (PC1 and PC2) from the PCA on SNP were incorporated as covariates.
- *e* is a random residual vector, distributed $e \sim \text{N}(0,\mathbf{I}{\sigma }_{e}^{2})$, with ${\sigma }_{e}^{2}$ signifying residual variance **I** the identity matrix.
- $u$ indicates a vector of random animal additive, dominance or overdominance genetic effects depending how the effects were calibrated for G. These effects are distributed as $u \sim \text{N}(0,\mathbf{G}{\sigma }_{u}^{2})$, where ${\sigma }_{u}^{2}$ is the modeled genetic variance.

The genomic relationship matrix G was constructed based on the method proposed by VanRaden (2008). It was computed as:

$$\mathbf{G}=\frac{\mathbf{Z}{\mathbf{Z}}^{{\prime }}}{2\sum {p}_{i}(1-{p}_{i})}$$

In this equation, Z is a centered genotype incidence matrix. We employed alternate calibrations of the genetic relationship matrix (**G**) to model different genetic effects within the population. The structure for the additive model is calibrated to represent genotype covariates as 0, 1, 2 where 0 = mm, 1 = Mm and 2 = MM (m = minor allele, M = major allele). For the dominance models, we adjust the calibration to reflect dominance effects. This structure is represented as 0, 1, 1 where 0 = mm, and 1 = Mm/MM, assuming dominance from the major allele. Alternatively, we assumed cases where the minor allele is dominant, represented by 1, 1, 0 where 1 = mm/Mm, 0 = MM. These calibrations were designed to capture complete dominance, where the heterozygote performs similarly to one of the homozygotes. The overdominance model is calibrated as 0, 1, 0 where 0 = mm/MM, 1 = Mm. This calibration is specifically designed to capture the heterozygote advantage, where the heterozygous genotype is assumed to have a superior phenotype compared to either homozygous form. In all calibrations, the major allele is denoted as the allele with the highest frequency in the population, and the minor allele is the alternative/least frequent allele in the population.

In the BOA GWAS, the **Z** matrix acts as a centered incidence matrix for BOA covariates. Therefore, BB denotes homozygous Brahman, AB stands for heterozygous variants, and AA represents homozygous Angus. For the additive model we calibrate **G** as 0 = BB, 1 = AB, 2 = AA. For the Angus dominance model the code is 0 = BB, 1 = AB, 1 = AA; for the Brahman dominance model the code is 1 = BB, 1 = AB, 0 = AA. Lastly, for the overdominance model, the code is 0 = BB, 1 = AB, 0 = AA.

### Genome-wide association studies

Single-trait GWAS were performed for HCW and MARB with SNP and BOA data using the weighted GBLUP (WGBLUP) [14]. The SNP/BOA effects and weights for additive, dominance, and overdominance effects were estimated with the WGBLUP method using `blupf90` and ‘postgsf90’ functions, which underwent three iterative processes. Under this approach, the influence of SNPs/BOA with greater effects becomes amplified, whereas the influence of markers with lesser effects diminishes.

Briefly, SNP effects and weights for the GWAS were derived as in Wang et al. [14] as follows:

1. Set the diagonal matrix of SNP variance or weights as identity, $\varvec{D}=\varvec{I}.$
2. Construct the **G** matrix: $\varvec{G} = \varvec{Z}\varvec{D}\varvec{Z}\varvec{{^\prime }} \varvec{\lambda }$, where $\lambda = 1/2 \sum {p}_{i}(1 - {p}_{i}).$
3. Predict GEBVs using GBLUP with blupf90 package.
4. Convert GEBVs to SNP effects ($\widehat{a}$) with postGSf90 package: $\widehat{a}= kDZ{^\prime }{G}^{-1}\widehat{u},$ where $\widehat{u}$ is the GEBV of genotyped animals.
5. Compute the weight for each SNP (${d}_{i}$) using a nonlinearA variance method: ${d}_{i}= {CT}^{\frac{\left|{\widehat{a}}_{i}\right|}{\sigma \left(\widehat{a}\right)} - 2}$, where CT is a constant for departure from normality equal to 1.05, $\left|{\widehat{a}}_{i}\right|$ is the estimated absolute SNP effect, and $\sigma \left(\widehat{a}\right)$ is the standard deviation of the vector of estimated SNP effects, with the maximum change in SNP variance limited to 10 [15, 16].
6. Normalize SNP weights to maintain the genetic variance constant.
7. Iterate from step 2, using the obtained weights to compute the G-matrix.

GWAS results are presented as the percentage of genetic variance explained by a sliding 10 kb window. The percentage of the direct genetic variance explained by a given SNP window was calculated according to [14]:

$$\frac{Var\left({w}_{i}\right)}{{\sigma }_{\mu }^{2}} \times 100= \frac{Var\left({\sum }_{j}^{B}{Z}_{j}\widehat{{a}_{j}}\right)}{{\sigma }_{\mu }^{2}}$$

where ${w}_{i}$ is the genetic value of the *i*^*th*^ 10 kb genomic window, *B* is the number of SNP within the *i*^*th*^ window, ${Z}_{j}$ is the vector of genotypes in the *j*^*th*^ SNP for all individuals, and $\widehat{{a}_{j}}$ is the estimated genetic effect for the *j*^*th*^ SNP within the *i*^*th*^ window. Genomic windows explaining over 1% of the genetic variance were deemed associated with the traits in question. We visualized our findings using manhattan plots, constructed with the R software [17]. For mapping SNPs to specific genes, we utilized Ensembl version 107 [18] and the UCSC ARS-UCD 1.2 genome assembly [19].

Certain markers explained over 1% of the genetic variation in both the additive and dominance GWAS analyses in either the SNP or BOA GWAS. To further refine our approach, we fit a linear model in base “stats” package in R [17] that incorporated both additive and dominance effects concurrently to mitigate potential confounding:

$$Trait= \mu +CG+PC1+PC2+Additive +Dominance + e$$

Where:

- *Trait* is the phenotype of interest (HCW or MARB).
- $\mu$ represents the overall mean across all observations.
- *PC1* and *PC2* represent continuous fixed effects of the first two principal components, adjusting for population structure and genetic background.
- *Additive* effect is modeled by categorizing genotype covariates linearly as 0, 1, 2, where, for the SNP GWAS, ‘0’ corresponds to homozygous for the minor allele (mm), ‘1’ to heterozygous (Mm), and ‘2’ to homozygous for the major allele (MM). Similarly, for the BOA GWAS, ‘0’ = BB, ‘1’ = AB, and ‘2’ = AA.
- *Dominance effect* is coded as 0, 1, 0, uniquely capturing the middle genotype’s effect by assigning ‘1’ to heterozygotes and ‘0’ to both homozygotes.
- *e* denotes the random error term.

Coding dominance in this manner is typically termed as “biological dominant” and is commonly used when accounting for additive and dominance effects simultaneously in genetic studies [20–23]. Using this methodology, we attempt to separate and estimate the additive and dominance effects of these markers explaining over 1% of the genetic variation in both the additive and dominance GWAS. Markers (SNP or BOA) that explained over 1% of the genetic variation in both the additive and dominance GWAS analyses were individually analyzed using this model. This allowed to discern that marker’s gene action on the trait of interest.

To ensure the findings from the BOA GWAS were intrinsic to the BOA genotypes and not confounded by SNP information, an analysis of variance (ANOVA) was conducted for each BOA marker that explained over 1% of the genetic variation. Models were ran with the base “stats” and “car” [24] packages in R [17]. To precisely attribute the observed genetic variation in the traits of interest to specific BOA genotypes, the following linear model was applied:

$$\begin{array}{l}Trait\, = \,\mu \, + \,CG\, + \,PC1\, + \,PC2\, + \\BOA\,Genotype\, + \,SNP\,Genotype\, + \,e\end{array}$$

Where:

- *Trait* is the phenotype of interest (HCW or MARB).
- $\mu$ represents the overall mean across all observations.
- *PC1* and *PC2* represent continuous fixed effects of the first two principal components, adjusting for population structure and genetic background.
- *BOA Genotype* is the fixed effect of the breed-origin allele genotype, considered categorical with three levels.
- *SNP Genotype* is the fixed effect of the SNP genotype, considered categorical with three levels.
- *e* denotes the random error term.

Subsequently, least square means (LSMeans) between BOA genotypic groups were obtained using the “emmeans” package [25] in R [17], aiming to isolate the impact of BOA genotypes while controlling for potential confounding SNP genotype effects.

## Results & discussion

### Phenotypes & genetic parameters

Table 1 presents the summary statistics for HCW and MARB in the Brangus cattle in this study. The dataset includes measurements from 1,043 animals for HCW and 1,050 animals for MARB. The average HCW was 373.05 kg with a standard deviation of 36.17 kg which is slightly below the national average of 390 kg documented in the 2016 National Beef Quality Audit [26]. The marbling scores varied from 210 to 850, with an average of 436. This aligns with previous studies by Lonergan et al. [27] who reported an average of 423, Phelps et al. [28] who reported an average of 445 in Brangus cattle, and the 2016 National Beef Quality Audit [26] which reported a national average of 475. Table 2 presents the genetic and residual variance components estimated using AIREML for SNP and BOA markers data. The variance explained by the additive model using SNP data showed substantial contribution of additive genetic variance for HCW and MARB, accounting for 34% and 52% of the phenotypic variation in HCW and MARB, respectively. These results corroborate previous moderate heritability estimates for HCW (0.57) and MARB (0.50) reported by Elzo et al. [29] from a multibreed Brahman-Angus cattle. When modeling dominance in HCW and MARB, there was an increase in the proportion of variance explained (Table 2). This indicates that when only additive effects were fit, non-additive components which were captured when modeling dominance were assigned to the residual. These estimates of genetic variance from SNP-based dominance models suggest that non-additive effects may also play a crucial role in determining these traits. Despite the smaller magnitude of BOA-based additive genetic variance estimates compared to SNP-based estimates, the percentage of phenotypic variation explained for HCW (0.14) and MARB (0.13) underscores the importance of the breed-specific additive component underlying these traits. Variance estimates from BOA-based dominance models showed breed-specific dominance effects only for the Brahman allele in MARB, as demonstrated by the increase in genetic variance estimate compared to the BOA-based additive model.

**Table 1:** Phenotypic data (number of animals, average, standard deviation, minimum and maximum value) for hot carcass weight (HCW) and marbling (MARB)

Trait	*N*	Mean	SD	Min	Max
HCW, kg	1043	373.05	36.17	253.56	505.30
MARB	1050	436.13	84.22	210.00	850.00

**Table 2:** Variance Components estimated using AIREML for hot carcass weight (HCW) and marbling score (MARB) for SNP and BOA models

Trait	Model	Marker information	Genetic variance (σ_µ_^2^)	Residual variance (σ_e_^2^)	Proportion of variance (σ_µ_^2^ / σ_µ_^2^ + σ_e_^2^)
HCW	Additive	SNP	356.38	697.77	0.34
HCW	Dominance Major allele	SNP	499.46	548.83	0.48
HCW	Dominance Minor allele	SNP	445.15	609.13	0.42
HCW	Overdominance	SNP	552.76	496.68	0.53
HCW	Additive	BOA	147.50	908.10	0.14
HCW	Dominance Brahman	BOA	133.47	917.36	0.13
HCW	Dominance Angus	BOA	134.98	915.39	0.13
HCW	Overdominance	BOA	78.38	964.88	0.08
MARB	Additive	SNP	3421.30	3169.80	0.52
MARB	Dominance Major allele	SNP	6125.80	419.54	0.94
MARB	Dominance Minor allele	SNP	3813.50	2749.30	0.58
MARB	Overdominance	SNP	4527.10	1986.20	0.70
MARB	Additive	BOA	845.51	5727.60	0.13
MARB	Dominance Brahman	BOA	915.27	5617.50	0.14
MARB	Dominance Angus	BOA	728.84	5836.20	0.11
MARB	Overdominance	BOA	275.20	6239.00	0.04

### Genome-wide association studies using SNP data

**Fig. 1:**
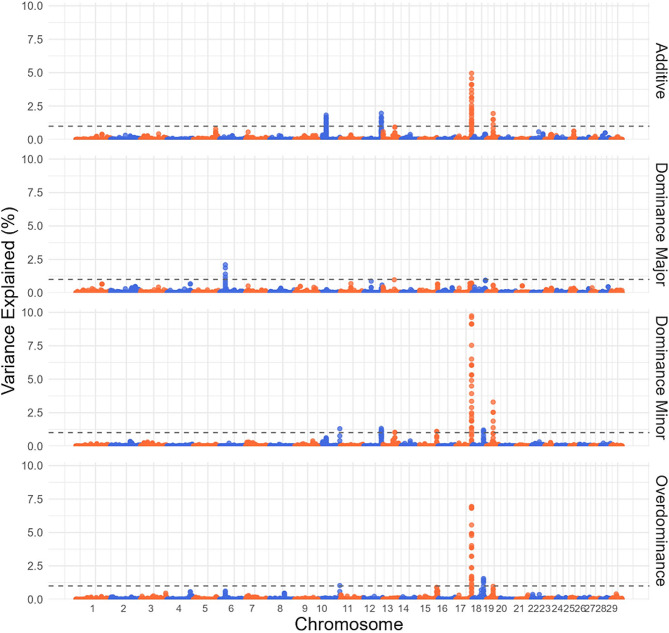
Manhattan plots for SNP GWAS on HCW. SNP GWAS modeling markers with additive, dominance from the major allele, dominance from the minor allele and overdominance effects on hot carcass weight, with significance thresholds indicating 1% of the genetic variance (grey dashed line). The variance explained by 10 kb genomic windows was estimated using single-trait WGBLUP using SNP information

Figure 1 presents the genetic variance explained by SNP effects for HCW in Brangus cattle, using a sliding 10 kb window across additive, dominance, and overdominance genetic models. A total of eight quantitative trait loci (QTLs) explaining more than 1% of the variance were identified across all four GWAS. Table 3 shows each QTL’s location, overlapping genes and variance explained, in addition *p*-values are reported in Additional file 2 Table S2.

**Table 3:** Genomic windows explaining more than 1% of the genetic variances from the SNP GWAS for HCW and MARB

Trait	Window				Variance Explained %			
	BTA	Start	End	Genes	Additive	Dom Major	Dom Minor	Overdominance
HCW	6	25,176,278	25,186,278	*ADH7*	-	2.10%	-	-
HCW	10	37,986,657	37,996,657	*STARD9*	1.82%	-	-	-
HCW	10	100,204,212	100,214,212	*PTPN21*	-	-	1.30%	1.03%
HCW	12	78,965,942	78,975,942	*CCDC168*	1.96	-	1.32%	-
HCW	15	78,377,630	78,387,630	*OR4C1J*	-	-	1.10%	0.89%
HCW	17	70,993,296	71,003,296	*ZNF280A, ZNF280B*	4.96	-	9.74%	6.94%
HCW	18	52,173,675	52,183,675	*ZNF227*	-	-	1.20%	1.56%
HCW	19	30,435,635	30,445,635	*DNAH9*	1.95	-	3.29%	-
MARB	1	20,716,311	20,726,311	*ENSBTAG00000053883*	-	1.17%	-	-
MARB	2	16,581,032	16,591,032		-	1.68%	-	-
MARB	5	40,358,402	40,368,402	*MUC19/BSM1, LRRK2*	-	4.51%	-	-
MARB	18	60,965,880	60,975,880	*LOC615600, NLRP12*	1.48%	2.13%	-	-
MARB	18	55,496,930	55,506,930	*NUCB1*	2.77%	1.00%	-	-
MARB	27	1,355,462	1,365,462	*ARHGEF10*	-	-	1.09%	-
MARB	28	5,870,881	5,880,881	*NTPCR, PCNX2*	1.52%	-	-	-

Columns show the trait, chromosome (BTA), the start and end location of the window in base pairs, overlapping genes (Genes) and the variances explained in the additive dominance (major and minor allele), and overdominance model

The most prominent QTL was on BTA17 (70,993,296–71,003,296), accounting for 4.96%, 9.74%, and 6.94% of the genetic variation under additive, dominance (minor allele), and overdominance models, respectively. This region has been previously reported to be associated with body weight and ribeye area in Angus beef cattle [30]. This QTL overlaps with the *Zinc Finger Protein 280 A* (*ZNF280A*) and *Zinc Finger Protein 280B* (*ZNF280B*) gene. *ZNF280A* is a transcription factor that has been associated with beef production and carcass quality traits in Hanwoo Korean cattle [31] and *ZNF280B* has been associated with carcass weight in Simmental beef cattle [32]. A notable QTL on BTA19 (30,435,635 − 30,445,635 bp) accounted for 1.95% and 3.29% of the genetic variation in the additive and dominance (minor allele) models, respectively. This region has been previously associated to body weight traits in Angus [30]. This QTL overlaps with the *dynein axonemal heavy chain 9* (*DNAH9*) gene, which encodes for the heavy chain subunit of axonemal dynein which attaches to microtubules and hydrolyzes ATP to mediate cilia and flagella movement. *DNAH9* has been previously linked to body measurement traits in pigs [33]. A peak on BTA10 (100,204,212 − 100,214,212 bp) explained 1.30% and 1.03% of the genetic variance under dominance (minor allele) and overdominance models, respectively. This region has been previously associated to carcass weight in Angus [30]. This region overlaps with the *Protein tyrosine phosphatase* (*PTPN21*) gene, a regulator of cell growth, differentiation, mitotic cycle, and oncogenic transformation, indicating its possible involvement in mechanisms influencing HCW. *PTPN21* is upregulated in pig breeds known for higher growth and muscling [34]. Research in humans has illustrated the role of *PTPN21* in growth and development. *PTPN21* activates the *Src* gene, which then interacts with a variety of signaling pathways, including the insulin-like growth factor (IGF-1) pathway [35, 36]. These pathways are essential for muscle growth and development and can potentially impact HCW. The peak on BTA15 explained 1.10% and 0.89% of the genetic variance under dominance (minor allele) and overdominance models, respectively. Previously in Angus this region has been associated to body height, body weight and longissimus muscle area [30]. This region overlaps the *olfactory receptor 4C1J* (*OR4C1J*) gene. *OR4C1J* is expressed in the olfactory epithelium, the nasal tissue responsible for odor detection. Recent research has revealed that olfactory receptors, despite their primary function in smell perception, might have pleiotropic effects. Connor et al. [37] provided reasonable evidence for a link between olfactory receptors and appetite regulation. If olfactory receptors influence metabolic processes or appetite regulation, they could indirectly impact an animal’s food intake and energy utilization, subsequently affecting body weight and carcass traits.

**Fig. 2:**
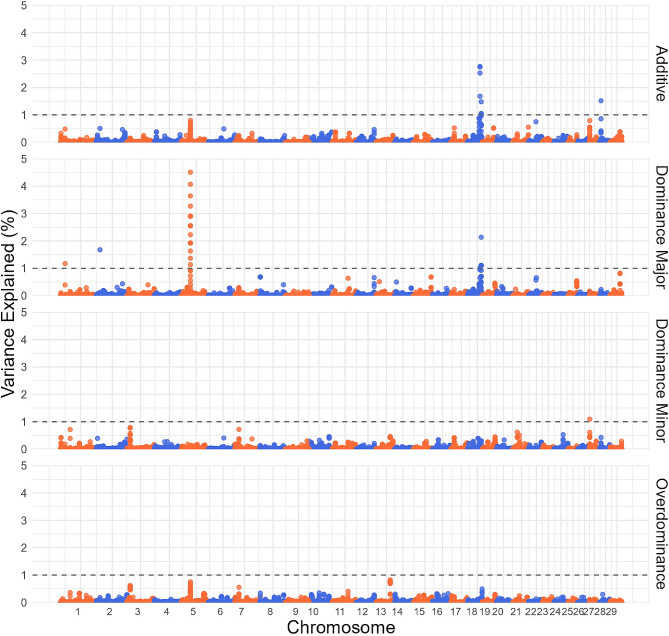
: Manhattan plots for SNP GWAS on marbling. SNP GWAS modeling markers with additive, dominance from the major allele, dominance from the minor allele and overdominance effects on marbling, with significance thresholds indicating 1% of the genetic variance (grey dashed line). The variance explained by 10 kb genomic windows was estimated using single-trait WGBLUP using SNP information

Figure 2 shows the genetic variance explained by SNPs effects for MARB using a sliding 10 kb window across additive, dominance, and overdominance genetic models. Table 3 displays the location of each QTL, the overlapping genes, and the explained variance. Additionally, Additional file 2 Table S3 contains the reported *p*-values associated with these findings. The QTL on BTA5 (40,358,402 − 40,368,402 bp) accounts for 4.5% of the variance under the dominance model (minor allele). This region has been previously associated to yield grade in cattle [38], which is primarily driven by marbling. This QTL is downstream from the *Leucine Rich Repeat Kinase 2* (*LRRK2*) gene, which has been previously associated with marbling in a multi-breed Angus and Brahman herd [39] and intramuscular fat content in hybrid pigs [40]. Another QTL on BTA18 (60,965,880 − 60,975,880 bp) was prominent in both the additive and dominance assuming the major allele GWAS. This QTL is located downstream of the *NLRP12* gene, which plays an important role in adipose tissue regulation, where diminished expression has been linked with obesity and increased fat accumulation in humans [41]. The QTL on BTA2 (16,581,032 − 16,591,032 bp) explains 1.68% of the variance under the dominance (major allele) model is downstream from the *CWC22* gene. *CWC22* has been associated to thicker backfat and better meat quality values in pig [42].

Results from the linear model analysis incorporating simultaneously additive and dominance effects are found in Additional file 2 Table S4A. The QTL on BTA17 overlapping the gene *ZNF280B* which explained the largest variation in the additive, dominance and overdominance models, showed a significant additive and dominance effect. The dominance effect resulted in a 10.47 kg reduction in HCW, and the additive effect had a 12.11 kg increase in HCW. All other overlapping QLTs for HCW showed significant additive effects. For MARB, the peak on BTA18 overlapping *NLRP12* showed a significant additive effect increasing MARB by 13.86 and a significant dominance effect resulting in an increase of 12.74. In both HCW and MARB overlapping peaks that explained a larger percent of the genetic variances in the additive models tended to exhibit an additive effect as expected.

### GWAS breed of origin allele

**Fig. 3:**
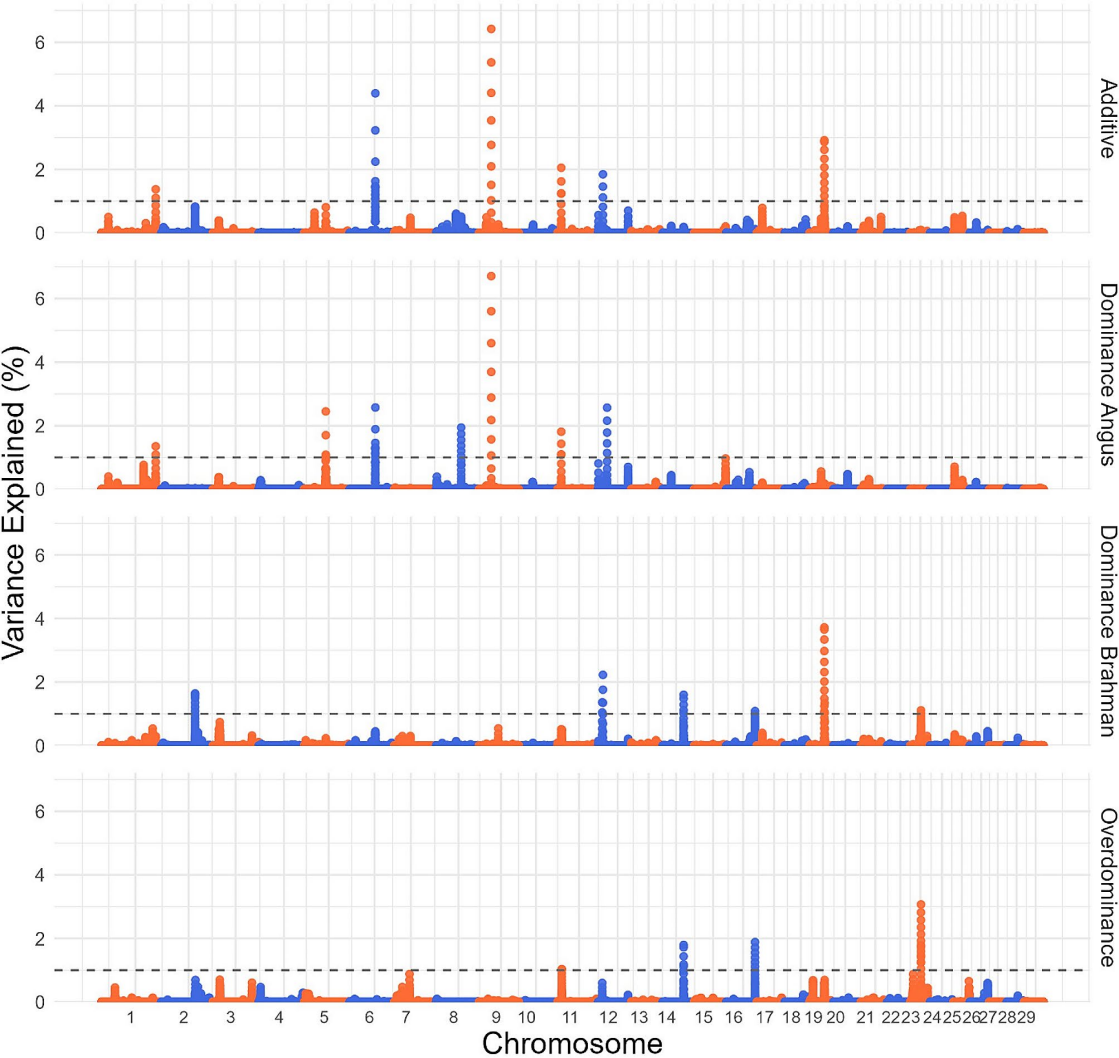
Manhattan plots for BOA GWAS on HCW. BOA GWAS modeling markers with additive, dominance from Angus, dominance from Brahman and overdominance effects on hot carcass weight, with significance thresholds indicating 1% of the genetic variance (grey dashed line). The variance explained by 10 kb genomic windows was estimated using single-trait WGBLUP using BOA information

Figure 3 displays the results from the BOA GWAS conducted on HCW using additive, dominance (Angus and Brahman) and overdominance models. Additionally, Additional file 2 Table S5 provides *p*-values for the BOA GWAS on HCW. Across all four GWAS models, a total of 13 QTLs were identified, each explaining more than 1% of the genetic variation in HCW. Notably, QTLs appear to be unique to the BOA GWAS and do not coincide with the SNP GWAS findings. Table 4 presents the details of these QTLs, including the genomic location, explained variance, associated genes, and least squares means adjusted for fixed effects and SNP genotype effects when significant. ANOVA results indicate only one notable effect from the SNP genotype on the QTL located on BTA8 (Additional file 2 Table S6). This suggests that the inheritance patterns captured by the BOA analysis provide additional insights into the variation in HCW, beyond what is explained by SNP genetic markers alone. Table 4 outlines the LSMeans of HCW for each BOA genotype across the studied QTLs, obtained from the ANOVA analysis.

**Table 4:** Genomic windows explaining more than 1% of the genetic variances from the BOA GWAS for HCW and corrected means for BOA genotypes

Window				Lsmeans (kg)			Variance Explained %			
BTA	Start	End	Genes	HCW - BB	HCW - AB	HCW - AA	Additive	Dom AN	Dom BRH	Overdominance
1	143,587,998	143,597,998	*SIK1*	^a^385 ± 3.63	375 ± 2.73	372 ± 2.29	1.37%	1.35%	-	-
2	89,088,722	89,098,722	*SG02*	386 ± 5.14	379 ± 2.66	^a^372 ± 1.99	-	-	1.64%	-
5	57,153,042	57,163,042	*SMARCC2,MYL6*	^a^370 ± 2.42	376 ± 2.77	378 ± 1.96	-	2.45%	-	-
6	67,879,766	67,889,766	*SGCB*	^a^369 ± 2.77	376 ± 3.6	376 ± 2.45	4.39%	2.57%	-	-
8	66,103,904	66,113,904	*CNTNAP3*	^a^371 ± 2.59	^b^381 ± 3.35	^ab^376 ± 1.81	-	1.94%	-	-
9	32,192,860	32,202,860	*MCM9*	^a^383 ± 3.65	367 ± 5.46	369 ± 5.82	6.42%	6.71%	-	-
11	9,261,903	9,271,903	*C11H2orf49,FHL2*	^a^381 ± 2.84	^ab^377 ± 3.27	^b^373 ± 2.87	2.05%	1.81%	-	-
12	12,438,276	12,448,276	*AKAP11*	373 ± 2.59	369 ± 3.26	^a^378 ± 2.54		-	2.23%	-
12	23,443,683	23,453,683	*FREM2*	^a^364 ± 4.58	^b^381 ± 4.78	^c^373 ± 3.39	-	2.57%	-	-
14	54,968,142	54,978,142	*PKHD1L1*	378 ± 11.9	374 ± 11.5	^a^366 ± 11.5	-	-	1.60%	1.79%
16	76,034,889	76,044,889	*ASPM*	372.79 ± 4.07	371.88 ± 2.37	^*^377.32 ± 1.73	-	-	1.08%	1.88%
19	38,984,113	38,994,113	*MRPL45,GPR179*	383 ± 3.82	383 ± 2.9	^a^372 ± 1.89	2.92%	-	3.72%	-
23	28,308,401	28,318,401	*MDC1*	374 ± 5.04	371 ± 3.41	375 ± 3.06	-	-	1.11%	3.07%

abc superscripts indicate groups that are significantly different at 0.05

Columns show the chromosome (BTA), the start and end location of the window in base pairs, overlapping genes (Genes), Lsmeans for hot carcass weight(HCW) in lb for the genotypic BOA combinations, where AA is homozygous Angus, AB is the heterozygote and BB is homozygote Brahman, and the variances explained in the additive, dominance AN (Angus) dominance BRH(Brahman) and overdominance models

The most pronounced effect was on BTA 9 (32,192,860 − 32,202,860 bp) and accounted for 6.4% and 6.7% of the genetic variation in HCW in the additive and Angus dominance GWAS models. The QTL overlaps with the *Minichromosome Maintenance 9 Homologous Recombination Repair Factor* (*MCM9*) gene. Mutations in *MCM9* are associated to a variety of diseases, where one of the symptoms is short stature [43], indicating this gene’s variations could lead to alterations in an animal’s height, potentially affecting carcass weights as a result. Contrary to expectation, the inheritance of the Angus BOA was associated with a decrease in HCW. Table 4 shows the LSMeans for HCW with the heterozygotes AB having an average HCW of 379.59 kg, homozygotes AA averaging 370.98 kg, and BB homozygotes averaging 374.15 kg, showing a partial dominance effect. Further analysis incorporating both additive and dominance simultaneously (Additional file 2 Table S4B) for *MCM9* indicated a trend towards significance resulting in a 6 kg decrease in HCW.

Another noteworthy peak was identified on BTA6 (67,879,766 − 67,889,766 bp), which contributed to 4.4% and 2.6% of the genetic variation in the additive and dominance Angus GWAS models. This QTL contains the *Sarcoglycan Beta* gene (*SGCB*). *SGCB*, a component of the sarcoglycan complex, is crucial for muscle integrity and contraction efficiency, and has been associated to growth traits in broiler chickens [44] and was also implicated in limb-girdle muscular dystrophy in humans [45]. Beta-sarcoglycan-deficient mice display progressive muscular dystrophy and muscular hypertrophy [46]. These results show the biological significance of *SGCB* related to muscling, indicating that variations within this gene might contribute to differences in an animal’s muscling. This, in turn, suggests that variations in *SGCB* could potentially influence the ultimate HCW of the animal due to its role in muscle-related processes. When incorporating both additive and dominance in the linear model, solely a significant additive effect was observed, indicating this region’s gene action is additive in nature (Additional file 2 Table S4B). This is somewhat expected since the variance explained in the additive model is larger than that from the dominance model for Angus.

A peak on BTA14 (54,968,142 − 54,978,142 bp) explained 1.6% and 1.79% of the variation in the dominant Brahman and overdominance GWAS models. This peak aligns with the *PKHD1L1* gene, exhibiting a positive and partial dominant influence on HCW when inherited from Brahman. However, no overdominance effect was observed, possibly due to a low number of homozygous BB individuals in this population.

A QTL on BTA19 (38,984,113 − 38,994,113 bp) explained 2.91% and 3.73% of the genetic variance in the additive and dominance Brahman GWAS. This region overlaps with the *GPR179* and *MRPL45* genes, showcasing a positive and complete dominant effect on HCW when inherited from Brahman. However, when incorporating both additive and dominance effects, there was no significant dominance effect (Additional file 2 Table S4B). A prominent peak on BTA12 (23,443,683 − 23,453,683 bp) accounted for 2.56% of the variance in the dominance Angus GWAS. This region overlaps with the *FREM2* gene, previously identified as a candidate gene for carcass traits in pigs [47]. Intriguingly, the heterozygote in this region seems to outperform both homozygotes, indicating a possible overdominance effect. Lastly, a peak on BTA23 (28,308,401 − 28,318,401 bp) explained 3.07% of the variation in the overdominance GWAS. This region harbors the *mediator of DNA damage checkpoint 1* (*MDC1*) gene, which has been associated with carcass traits in pigs [48]. In this case, the heterozygotes (AB) appear to underperform in comparison to both homozygotes.

**Fig. 4:**
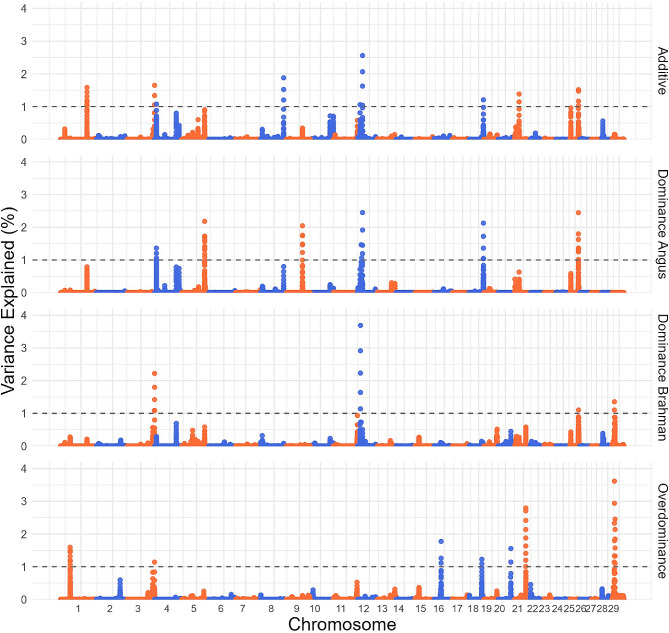
Manhattan plots for BOA GWAS on MARB. BOA GWAS modeling markers with additive, dominance from Angus, dominance from Brahman and overdominance effects on marbling, with significance thresholds indicating 1% of the genetic variance (grey dashed line). The variance explained by 10 kb genomic windows was estimated using single-trait WGBLUP using BOA information

Figure 4 illustrates the results from the BOA GWAS conducted on MARB using an additive, dominance (Angus and Brahman) and overdominance. Additionally, Additional file 2 Table S7 provides *p*-values for the BOA GWAS on MARB. A total of 18 QTLs were identified across all four GWAS, each explaining more than 1% of the genetic variation in MARB. These peaks do not coincide with the peaks from the SNP GWAS on MARB, indicating potentially different underlying genetic mechanisms. Table 5 shows the QTLs location, variation explained, overlapping genes and least square means for the BOA genotypes when adjusted for fixed effects and SNP genotype effects if significant. Results from our ANOVA analysis indicate no significant SNP effect for the BOA QTLs of interest, except for the BOA QTL on BTA12 (21,552,338–21,562,338 bp) which had a significant SNP effect (Additional file 2 Table S8). Table 5 outlines the least square means of MARB for each BOA genotype across the studied QTLs, obtained from the ANOVA analysis. It is noteworthy that certain QTLs lacked significant differences between BOA genotypic combinations in the ANOVA, likely due to substantial standard errors associated with one or more of the genotypes. However, the mean differences were still considerable, which contributed to their detection in the GWAS analysis.

**Table 5:** Genomic windows explaining more than 1% of the genetic variances from the BOA GWAS for MARB and corrected means for BOA genotypes

Window				Lsmeans			Variance Explained %			
BTA	Start	End	Genes	MARB - BB	MARB - AB	MARB - AA	Additive	Dom AN	Dom BRH	Overdominance
1	42,125,147	42,135,147	*CRYBG3*	437 ± 6.53	449 ± 7.42	437 ± 7.93	-	-	-	1.59%
1	116,639,978	116,649,978	*IGSF10*	443 ± 20.30	414 ± 89.9	417 ± 38.90	1.58%	-	-	-
3	120,425,644	120,435,644	*HDLBP*	447 ± 7.78	449 ± 6.19	^a^434 ± 5.08	1.65%	-	2.22%	-
4	7,520,343	7,530,343	*ABCA13*	446 ± 5.18	431 ± 10.99	436 ± 4.55	1.08%	1.36%	-	-
5	100,711,417	100,721,417	*OVOS2*	^a^452 ± 6.35	435 ± 7.21	434 ± 9.11	-	2.18%	-	-
8	101,136,885	101,146,885		443 ± 20.30	414 ± 89.90	417 ± 38.90	1.88%	-	-	-
9	70,865,195	70,875,195	*VNN1,VNN2,VNN3*	^a^422 ± 7.31	447 ± 7.60	437 ± 6.86	-	2.04%	-	-
12	12,438,276	12,448,276	*AKAP11*	428 ± 6.48	428 ± 8.13	^a^444 ± 6.37	-	-	3.69%	-
12	21,552,338	21,562,338	*NEK3*	406 ± 13.30	423 ± 22.6	417 ± 14.0	2.56%	2.45%	-	-
16	30,575,226	30,585,226	*KIF28*	437 ± 6.62	^a^454 ± 6.90	435 ± 8.15	-	-	-	1.77%
18	56,335,937	56,345,937	*ZNF473*	427 ± 10.94	447 ± 8.04	437 ± 9.42	-	-	-	1.23%
18	63,418,581	63,428,581	*NLRP13*	^a^441 ± 20.10	424 ± 20.50	422 ± 20.40	1.21%	2.13%	-	-
20	55,278,018	55,288,018		436 ± 10.58	^a^456 ± 7.12	436 ± 6.01	-	-	-	1.55%
21	49,193,212	49,203,212	*MIA2*	^ab^442 ± 8.66	^b^432 ± 5.62	^a^446 ± 5.14	-	-	-	2.80%
21	19,644,859	19,654,859		^a^431 ± 5.78	447 ± 7.69	445 ± 5.42	1.38%	-	-	-
25	36,972,814	36,982,814	*PTCD1*	^a^463 ± 10.01	^ab^449 ± 11.50	^b^433 ± 9.64	1.52%	1.33%	1.10%	-
25	36,530,556	36,540,556	*CYP3A28*	439 ± 6.89	427 ± 8.94	442 ± 6.12	-	2.45%	-	-
29	9,824,574	9,834,574	*SYTL2*	^ab^441 ± 13.99	^a^428 ± 8.04	^b^449 ± 6.60	-	-	1.35%	3.62%

abc superscripts indicate groups that are significantly different at 0.05

Columns show the chromosome (BTA), the start and end location of the window in base pairs, overlapping genes (Genes), Lsmeans for marbling (MARB) for the genotypic BOA combinations, where AA is homozygous Angus, AB is the heterozygote and BB is homozygote Brahman, and the variances explained in the additive, dominance AN (Angus) dominance BRG(Brahman) and overdominance models

The peak on BTA3 (120,425,644–120,435,644 bp) explains 1.65% and 2.22% of the genetic variation in the additive and Brahman dominance GWAS. This region overlaps the gene encoding for the *high density lipoprotein binding protein* (*HDLBP*), which is a candidate gene for intramuscular fat in pigs [49]. *HDLBP* was seen to be upregulated in tender meat in Nellore cattle [50]. Interestingly this region saw an increase in marbling with the inheritance of the Brahman BOA, exhibiting a complete dominance effect. The peak on BTA9 (70,865,195 − 70,875,195 bp) explained 2.04% of the variance explained in the Angus dominance GWAS. This region overlaps three *vanin* genes (*VNN1,VNN2,VNN3*), mutations in *VNN1* have been linked to fatty acid composition changes in Japanese cattle [51]. *VNN1* is known to encode an enzyme critical for pantetheine breakdown, a precursor necessary for fatty acid synthesis [52]. This QTL has a complete dominant effect or overdominance effect where an increase in marbling was associated with the inheritance of Angus BOA. The QTL on BTA29 explains 1.35% and 3.62% of the genetic variance in the Brahman dominance and overdominance models. This QTL coincides with the *Synaptotagmin-like protein 2* (*SYTL2*) gene, noted for differential expression in Nellore cattle with varying marbling score [53], indicating its potential role in marbling traits. This gene has a seemingly overdominance effect where the heterozygote underperformed compared to both homozygotes. Lastly, the peak on BTA21 (49,193,212 − 49,203,212 bp), which explains 2.80% of the genetic variation in the overdominance GWAS model, is located near the *MIA2* gene. *MIA2* is implicated in regulating cholesterol metabolism and thus may influence cellular fat storage [54]. In this region, an overdominance effect is observed where the heterozygote BOA genotype appears to be less favorable compared to the homozygous genotypes.

Several markers overlapped in either the additive and dominance for Angus or Brahman GWAS, these markers were further investigated by fitting a linear model simultaneously modelling both additive and dominance effects. All of these overlapping QTLs had a significant additive effect, and no significant dominance effects (Additional file 2 Table S4B). In general, many of these QTLs had large dominance effects but they also had large standard deviations leading to a lack of significance.

### Implications

This study expands the catalog of known QTLs for HCW and MARB, reaffirming the roles of genes such as *ZNF280B* and *LRRK2*, while also highlighting novel associations with the *DNAH9*, *ADH7* and *CWC22* genes, which accounted for a substantial proportion of the genetic variation. Results from both SNP and BOA GWAS on HCW and MARB illustrated how the additive models captured many dominance-influenced QTLs, and the dominance models captured many additive-influenced QTLs, seen in Additional file 2 Table S4A and S4B. These results align with previous research and theory on additive and dominance variation, where modeling either additive or dominance gene actions can capture variance explained by the each other [55]. The modeling of dominance in this study has unveiled certain QTLs not identified by the additive model alone, underscoring the necessity of considering non-additive genetic effects. The QTL on BTA6 overlapping the *ADH7* gene was solely seen when modeling for a dominance effect for HCW and the QTL overlapping BTA18 for the *LRKKS2* gene was solely seen when modeling for a dominance effect for MARB. These QTLs underscore the importance of considering non-additive genetic effects in genetic analyses of composite breeds, where additive models can fail to identify certain QTLs exhibiting dominance. This may explain why the estimated genetic variances from the dominance models explained a higher proportion of the variance compared to the additive models in the SNP GWAS for MARB and HCW seen in Table 2. Certain QTLs showing a significant dominance effect also explained a large percentage of the genetic variation such as the QTL overlapping *ZNF280B* and *NLRP12*, leading credence to the importance of non-additive effects for MARB and HCW.

Integrating BOA into genomic evaluations aims to enhance the accuracy of estimating SNP effects, especially in composites and crossbred cattle characterized by diverse genomic structure. The LD between genomic markers and QTLs can differ in the purebred parents, integrating BOA allows for differences in estimated SNP effects depending on the BOA of the marker. Previous studies across multiple species testing BOA inclusion in genomic prediction of crossbreed animals have shown varying resulting, showing small increases in accuracy or no increases in accuracy [4–6, 56]. However, several studies have shown that employing BOA methods can mitigate bias in genomic prediction estimates and improve the accuracy of estimating specific SNP effects [57, 58]. In this study we further attempted to identify key QTLs effect MARB and HCW using solely BOA information.

By employing a BOA GWAS approach several QTLs associated with MARB and HCW in Brangus cattle were identified. Significantly, the QTLs identified do not overlap with those in SNP GWAS, indicating that BOA may harbor genomic information that goes beyond what is captured by SNPs alone. These discrepancies could stem from the challenge of precisely assessing SNP effects in composite populations. Variation in LD patterns and allele frequencies between parental breeds could potentially mask the effects of specific QTLs. Another potential factor could be inherited differences in gene expression stemming from the distinct purebred backgrounds, consequently impacting the traits under investigation. The BOA GWAS may be particularly sensitive to detecting heritable gene expression differences as it is a better indicator of breed inheritance compared to SNPs. This is highlighted by the QTLs overlapping the *HDLBP* and *SYTL2* genes which are known to influence marbling at the gene expression level in Nellore cattle [49, 53], another *Bos taurus indicus* breed. This suggests that these genes have a similar role in the Brahman breed and contribute to the phenotypic variations in Brangus cattle. The dominance effects identified in BOA QTLs offer an avenue for targeted mating plans within the Brangus breed, aiming to optimize heterosis and maintain genetic diversity. By carefully selecting mates based on these BOA QTLs, breeders can enhance beneficial traits while preserving the breed’s genetic variability. Such an approach not only contributes to the sustainability of the herd but also supports the development of a more productive and resilient Brangus population.

Our study also reveals a positive impact of specific Brahman haplotypes on HCW on BTA11, and BTA18 and MARB on BTA3 and BTA18, suggesting that the introgression of Brahman QTLs can impart advantageous traits for beef production contrary to popular belief. These findings highlight the potential of exploiting breed-specific genetic variation to improve production traits. The identification of these QTLs opens new avenues for selective breeding, enabling new selection opportunities for enhanced meat quality and desirable carcass characteristics in Brangus cattle. Utilizing BOA appears to be a more effective method for identifying regions that contribute to heterosis, as these regions originate from different breeds. This is supported by the diverse number of QTLs exhibiting a spectrum of dominance effects, including partial, complete, and overdominance identified with the use of BOA GWAS. These findings highlight the potential for genetic improvement within composite breeds by exploiting dominance to retain and capitalize on the production benefits derived from heterosis. This study lays the groundwork for further research aimed at validating the identified QTLs across diverse Brangus populations and investigating the biological mechanisms underlying the effects on MARB and HCW. However, despite the identification of several QTLs through BOA GWAS, there remains sparse literature on the functional implications of these genes for the traits of interest in beef cattle. This gap signifies an opportunity for future studies to explore the molecular pathways and gene networks impacted by these QTLs, thereby providing a more detailed understanding of their role in carcass and meat quality traits.

## Conclusion

The insights gained from this research not only advances our understanding of Brangus cattle genetics but also reinforce the importance of acknowledging and harnessing the genetic complexity inherent in composite breeds. This understanding is pivotal for designing genetic improvement strategies that capitalize on the unique genetic resources embedded in breed combinations. With the use of BOA, we enhance our ability to estimate SNP effects for achieving additive genetic gain. Moreover, we can more effectively identify regions that contribute to heterosis by evaluating the dominance value. Allowing for better mating decisions to increase productivity whilst maintaining genetic diversity for a more profitable and healthier herd. Moving forward, there is a critical need for integrated research approaches that combine genomic analyses with functional studies to elucidate the contribution of these QTLs to the phenotypic variability in cattle.

### Electronic supplementary material

Below is the link to the electronic supplementary material.

**Supplementary Material 1**: GWAS Manhattan plots based on *p*-values.: Supplementary Figs. 1–4 - Manhattan plots of pvlaues from SNP and BOA GWAS modeling additive, dominance and overdominance effects on hot carcass wieght and marbling

**Supplementary Material 2**: Tables of variance component estimates, significant markers from GWAS and ANOVA results. Supplementary Tables 1 – Esimtates of variance compoents from additive SNP and BOA models with and without including PCQ and PC2 as fixed covariates. Supplementary Tables 2–3 - *P*-values for significant markers from the SNP GWAS on HCW and MARB. Supplementary Tables 4 – Dominance and additive effects of overlapping QTLs in the additive and dominance GWAS for both SNP and BOA GWAS in HCW and MARB. Supplementary Tables 5 – *P*-values for significant markers from the BOA GWAS on HCW. Supplementary Tables 6- ANOVA results from markers explaining over 1% of the genetic variance in the BOA GWAS for HCW. Supplementary Tables 7 – *P*-values for significant markers from the BOA GWAS on MARB. Supplementary Tables 8- ANOVA results from markers explaining over 1% of the genetic variance in the BOA GWAS for MARB

## Data Availability

The datasets generated and/or analyzed during the current study are available in the European Variation Archive repository, PRJEB75981. Scripts used for analysis are available on Github https://github.com/gzayasPR/BOA_GWAS.
