# Supplementary material for: Breed of origin analysis in genome-wide association studies: enhancing SNP-based insights into production traits in a commercial Brangus population
Source: BMC Genomics. 2024 Jul 1;25:654. doi: 10.1186/s12864-024-10465-1 (PMC11218112; doi:10.1186/s12864-024-10465-1)

Supplemental Figure 1: Manhattan plots of pvlaues from SNP GWAS modeling additive, dominance and overdominance effects on hot carcass weight (HCW), with significance thresholds using an FDR of 0.05 (grey dashed line).
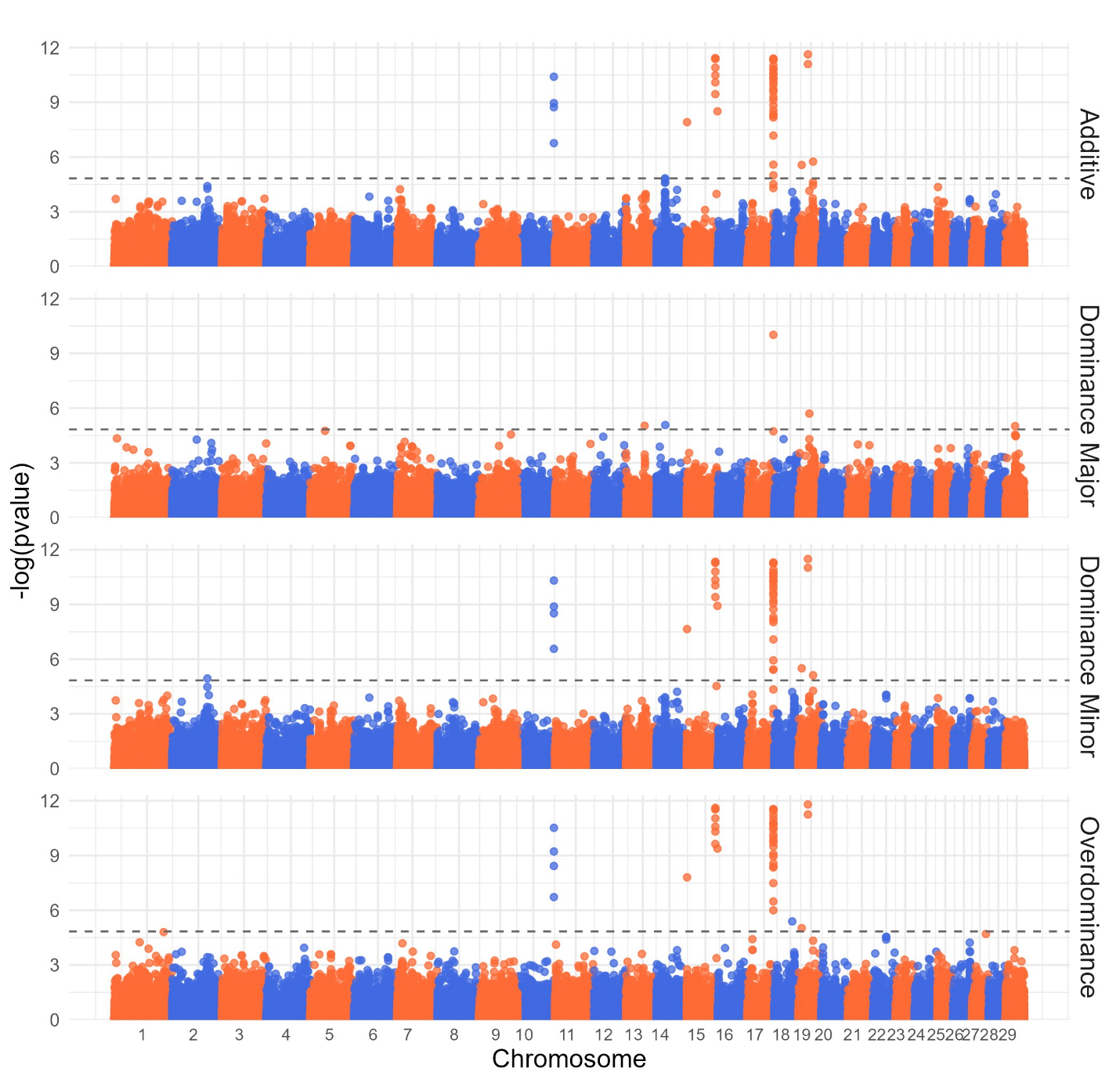


Supplemental Figure 2: Manhattan plots of pvlaues from SNP GWAS modeling additive, dominance and overdominance effects on marbling (MARB), with significance thresholds using an FDR of 0.15 (grey dashed line).
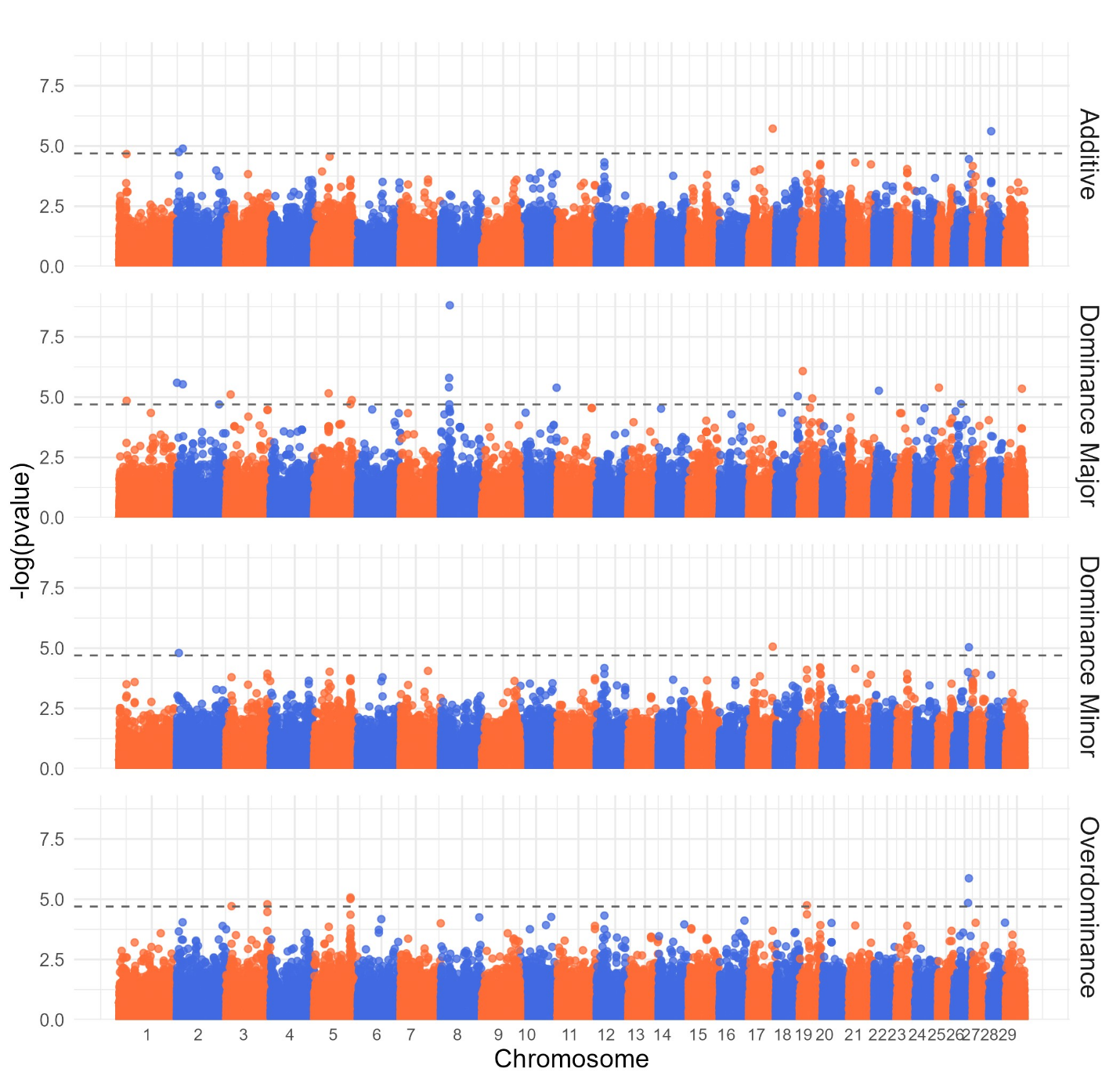


Supplemental Figure3: Manhattan plots of pvlaues from BOA GWAS modeling additive, dominance from Angus, dominance from Brahman and overdominance effects on hot carcass weight (HCW), with significance thresholds using an FDR of 0.30 (grey dashed line).


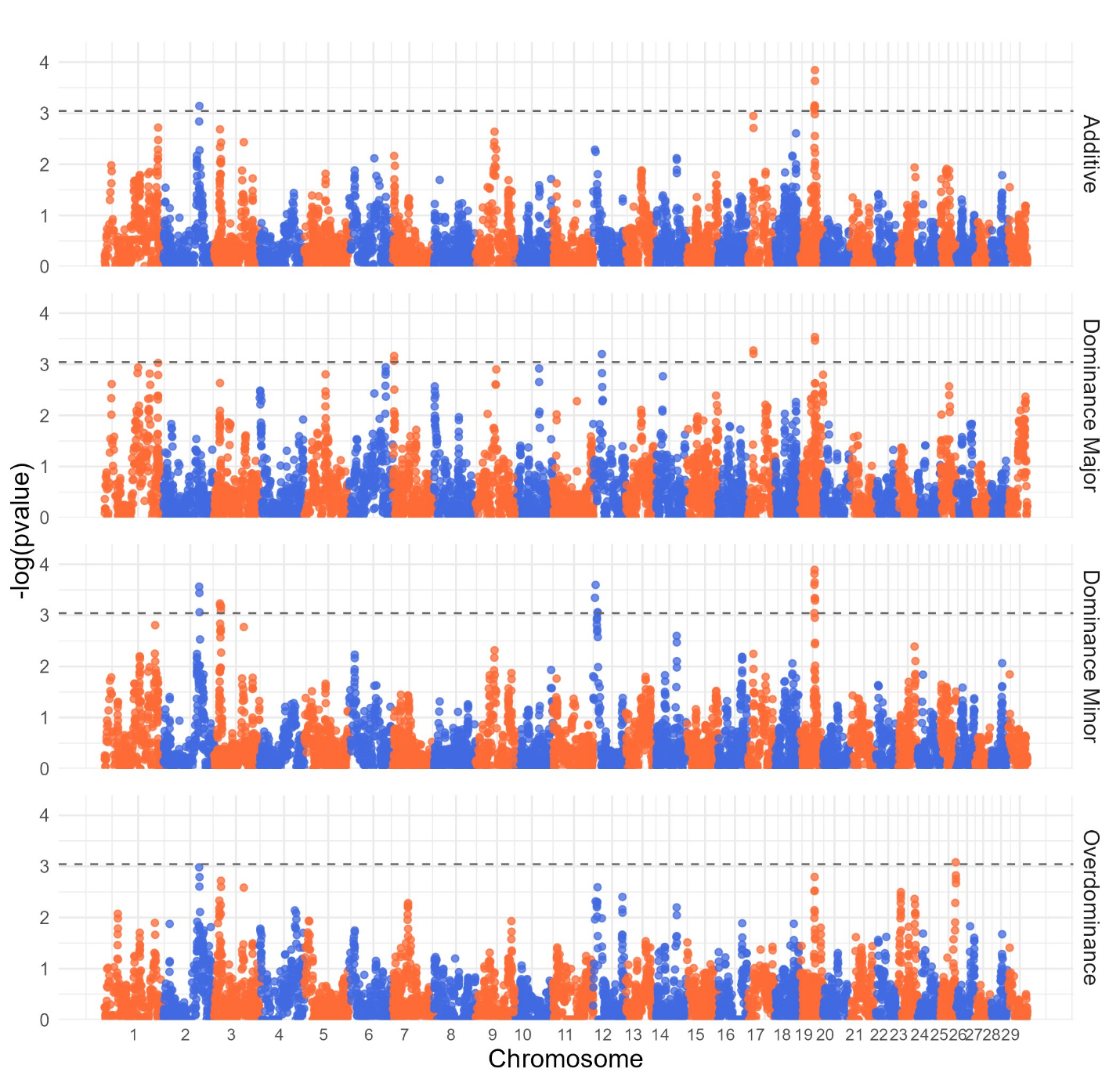


Supplemental Figure4: Manhattan plots of pvlaues from BOA GWAS modeling additive, dominance from Angus, dominance from Brahman and overdominance effects on marbling(MARB), with significance thresholds using an FDR of 0.175 (grey dashed line).


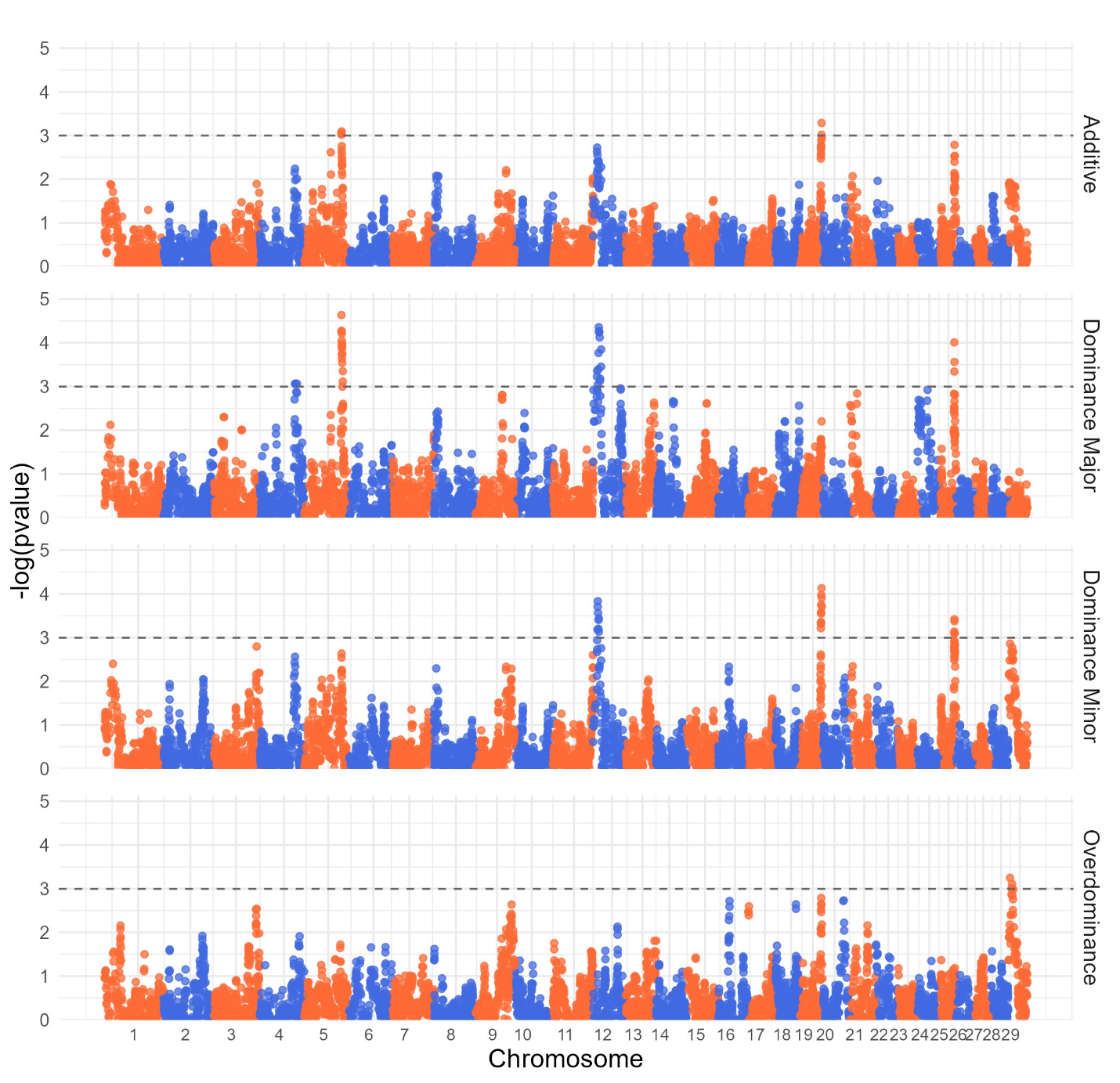

Supplement: Supplementary file 1 — Supplementary Material 1: GWAS Manhattan plots based on p-values.: Supplementary Figs. 1–4 - Manhattan plots of pvlaues from SNP and BOA GWAS modeling additive, dominance and overdominance effects on hot carcass wieght and marbling [file 12864_2024_10465_MOESM1_ESM.docx]
